# Real-Time Surface EMG Pattern Recognition for Hand Gestures Based on an Artificial Neural Network

**DOI:** 10.3390/s19143170

**Published:** 2019-07-18

**Authors:** Zhen Zhang, Kuo Yang, Jinwu Qian, Lunwei Zhang

**Affiliations:** 1 School of Mechatronic Engineering and Automation, Shanghai University, Shanghai 200444, China; 2 School of Aerospace Engineering and Mechanics, Tongji University, Shanghai 200092, China

**Keywords:** surface electromyography, artificial neural network, real-time, gesture recognition

## Abstract

In recent years, surface electromyography (sEMG) signals have been increasingly used in pattern recognition and rehabilitation. In this paper, a real-time hand gesture recognition model using sEMG is proposed. We use an armband to acquire sEMG signals and apply a sliding window approach to segment the data in extracting features. A feedforward artificial neural network (ANN) is founded and trained by the training dataset. A test method is used in which the gesture will be recognized when recognized label times reach the threshold of activation times by the ANN classifier. In the experiment, we collected real sEMG data from twelve subjects and used a set of five gestures from each subject to evaluate our model, with an average recognition rate of 98.7% and an average response time of 227.76 ms, which is only one-third of the gesture time. Therefore, the pattern recognition system might be able to recognize a gesture before the gesture is completed.

## 1. Introduction

Hand gestures are one type of communication. Gesture recognition provides a smart, natural, and convenient human–computer interaction (HCI) approach. It is an important part of HCI and has a wide range of applications in engineering and intelligent devices. It shows great potential in the control of bionic hands [1], virtual game control [2], sign language translation [3], and intelligent robotics [4].

There are many sensors used in hand gesture recognition for data acquisition, including cameras [5,6,7,8], cyber gloves [9,10], surface electromyography (sEMG) [11,12], and radio frequency [13]. Kundu et al. [14] combined the signals of the inertial measurement unit and sEMG to infer the movement of hands and fingers. Taylor et al. [7] proposed a new real-time hand tracking system based on a single depth camera, which can accurately reconstruct the complex joint posture of the hand. Microsoft’s Kinect sensor is used to obtain depth images and bone information to identify gestures in [15]. A new sensing technology that uses miniature radar to detect touchless gesture interactions has been developed by Google [16]. Based on the types of sensors mentioned above, the sEMG sensors can be applied for hand gesture recognition because they are not affected by the variations of light, position, and orientation of the hand.

Machine learning is used to solve the problem of hand gesture recognition based on sEMG signals. The most common classifiers for hand gesture recognition include support vector machines [17,18], k-nearest neighbors (k-NN) [11,12], decision trees [19], random forest [20], linear discriminant analysis [21,22], artificial neural network (ANN) [23,24], convolutional neural networks [25,26], and gated recurrent unit network [27]. The conventional features used for hand gesture recognition are defined in the following domains: time domain, such as mean absolute value (MAV) and zero crossing (ZC) [28]; frequency domain, such as median frequency and power spectrum ratio [29]; and time-frequency domain, such as wavelets [30]. Models based on these classifiers and feature domains present high recognition accuracy. However, most of them have to wait for the completion of the gestures and cannot be applied in a real-time system. Moreover, some researchers have investigated the featureless approach that directly feeds the preprocessed data without feature extraction into a classifier, a process which may reduce the computational cost [31,32]. However, extracting appropriate features from processed data can strengthen the inherent characteristics of the sEMG signal, of which the feature selection is the key to improve the discrimination performance. Therefore, hand gesture recognition is still an open subject for new research.

Many applications for gesture recognition, such as prothetic limb control, often require real-time response, which means that the total time available for signal acquisition and pattern recognition cannot exceed 300 ms [33]. There is a challenge in designing a real-time gesture recognition system that has both low computational cost and excellent performance. Recently, researchers have used sEMG signals to design real-time gesture recognition systems. A real-time locomotion mode recognition method based on a transformed correlation feature analysis is proposed in [11] which can be completed in 264.49 ms, including 256 ms required for sEMG signal collection. Lu et al. [34] proposed a myoelectric pattern recognition scheme that uses four channels of sEMG signals to detect and identify the user’s intentions for six different hand movements and then drives the exoskeleton to help the user complete the desired motion. The total hysteresis of the system is approximately 250 ms. Crepin et al. [22] proposed that the use of linear discriminant analysis to perform sEMG pattern classification and 13 hand motions can be identified with an updated prediction every 192 ms. However, in these studies, they used fixed time intervals to detect the gesture which may not be the best values for improving the eventual recognition accuracy. Further study of selecting preferred time intervals is required.

In this paper, we propose a new gesture recognition model based on ANN and sEMG signals to achieve real-time response. For data acquisition, we use the sEMG signal measured by an MYO armband to identify a set of five gestures. For preprocessing, we use a low pass filter to remove noise and smooth the signal. For feature extraction, we use the preprocessed signals in the sliding window and five time-domain features to form the feature vector. For classification, we apply an ANN algorithm to label the observation of each sliding window, and then adopt the trained classifier to give the recognition result during the action of the gesture. 

The main contributions are as follows:

(1) The proposed method is a gesture recognition method which can not only recognize the gesture in real time, but also has high recognition accuracy.
(2) The main parameters, such as sliding window size and threshold of activation times, are analyzed.

The rest of this article is organized as follows. Section 2 describes the sensor used in this work and the process of data acquisition. Section 3 introduces the proposed method in detail. Section 4 presents the experimental results and analysis. In addition, Section 5 summarizes the paper.

## 2. Sensors and Data Acquisition

**Figure 1:**
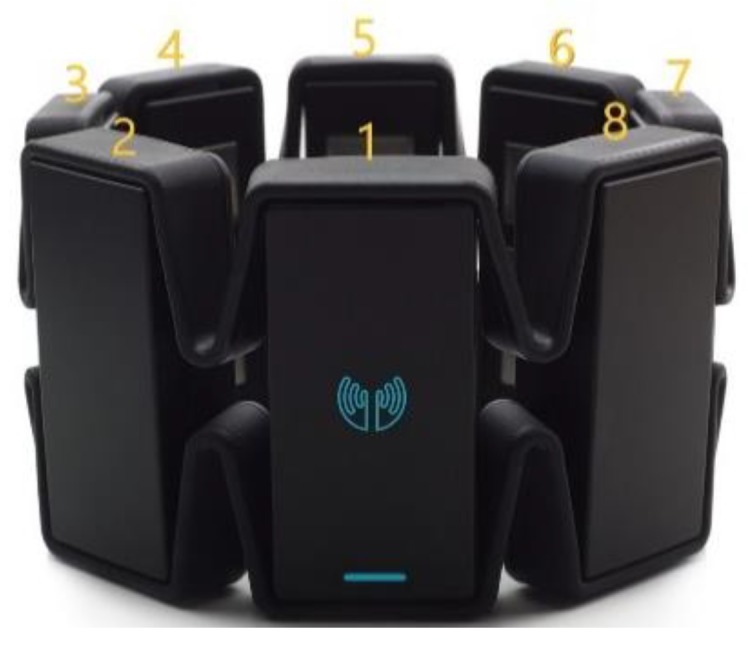
The armband for data acquisition.

To facilitate real-time processing, we use the MYO armband (Thalmic Labs, Waterloo, Canada) to acquisition of sEMG data [32]. The MYO armband is composed of eight sEMG dry sensors (as shown in Figure 1). These sensors measure the electrical activity of the muscles of the forearm at a sampling rate of 200 Hz with 8 bits of resolution for each sensor. Data from all of these sensors is transmitted to the computer via Bluetooth. Additionally, the armband is also capable of measuring angular velocity, acceleration, and orientation of input axes by means of a built-in inertial measurement unit. In this study, we merely take advantage of the sEMG portion.

**Figure 2:**
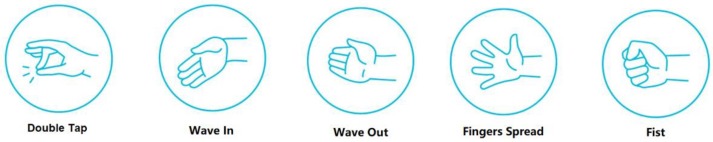
Five gestures.

A total of 12 healthy subjects (eight males and four females, aged from 22 to 26, all right-handed) volunteered for this study. Subjects were not trained before the test. At the beginning of the experiment, each subject sat in a comfortable chair and relaxed his/her arm. Then they were asked to naturally perform five gestures with randomized order: Fist, Wave In, Wave Out, Fingers Spread, and Double Tap (as shown in Figure 2). In the training set, there were five repetitions of the five gestures recorded during two seconds. In the testing set, there were 30 repetitions recorded during five seconds of the five gestures. For every repetition, the subject started with his/her arm relaxed, then performs the gesture, and then returns the arm to the relaxed position until the end of the recording.

## 3. Method

### 3.1. Overview

**Figure 3:**
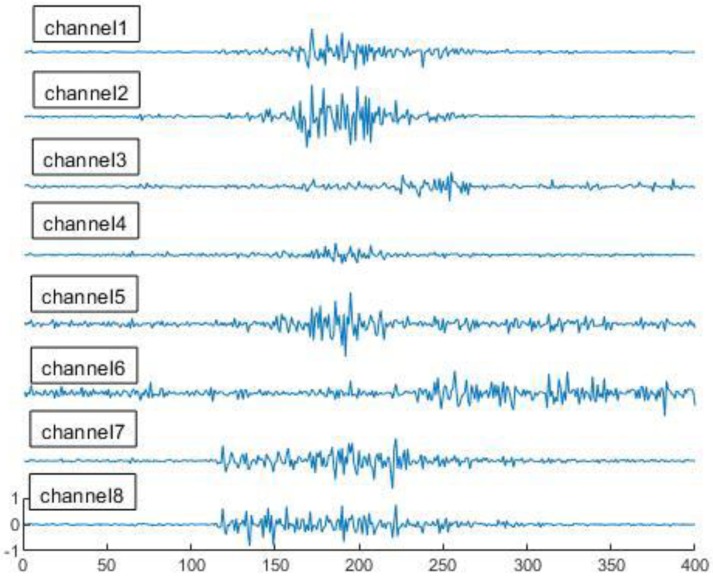
Original surface electromyography (sEMG) signals recorded by the MYO armband.

In this section, we describe the structure of the proposed model. The original signal (as shown in Figure 3) is preprocessed for rectification and filtered to remove the noise at first. Then, the representative time domain features are extracted. An ANN classification approach combining all the features is proposed. The algorithms of the training and testing in our model are presented separately in detail.

### 3.2. Training Part

#### 3.2.1. Preprocessing

**Figure 4:**
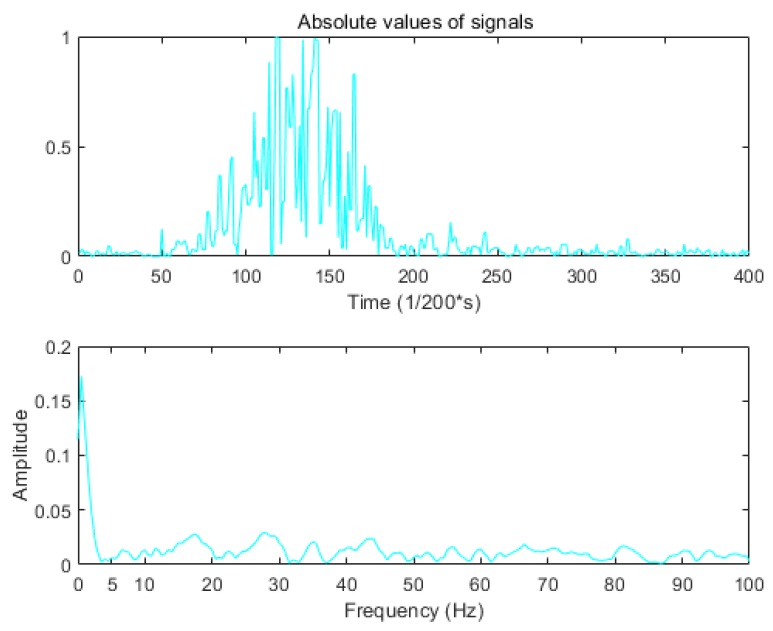
Fourier transform for the absolute values.

The purpose of the preprocessing is to denoise the acquired signal and make it easy to extract features. The original signal has some additional noise that can generate invalid features and interfere with the classification. For training, the observed signals are normalized at first, with each element of each matrix T=(T1,T2⋯T8) being in the range [−1, 1]. The original signal in each channel is then rectified using an absolute value function. In order to smooth the signal and reduce the noise, we design the filter by analyzing the signal frequency component and noise. Using the Fourier transform, we can find that the cut-off frequency set at 5 Hz is reasonable, as shown in Figure 4. Thus, we use the 4th order digital Butterworth filter whose cut-off frequency is 5 Hz. 

Then, we use the muscle detection function described in [14] to remove the head and tail that refer to the relaxed position for extracting the muscle activity range of every repetition in the training set. Meanwhile, we attempt to determine the muscle activity region by computing the spectrum energy from filtered data, and all the spectrum energy points according to sampling interval greater than a certain predefined empirical threshold (20 dB/Hz) can be extracted. Thus, we can detect the time area of muscle activity.

#### 3.2.2. Feature Extraction

**Figure 5:**
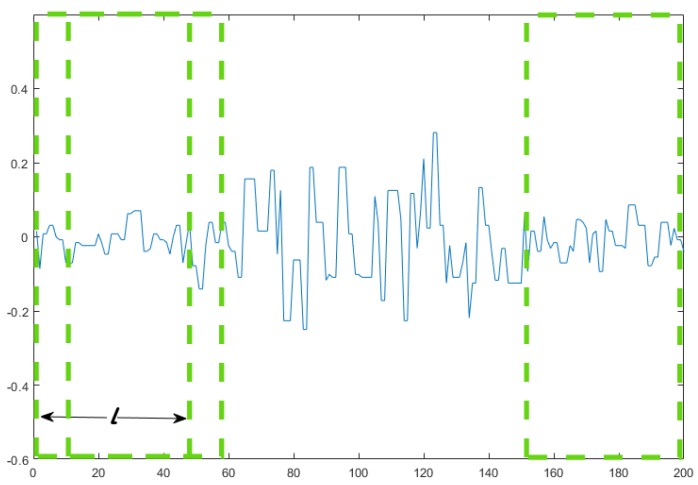
The sliding window extracts signal segments of muscle activity regions on one channel.

We use the sliding window technique for feature extraction in the proposed model. The data set is divided into data segments. We use the length *l* sliding window to divide the data as shown in Figure 5. For high precision in real time, the stride size of the two consecutive sliding windows is set to one point (5 ms). In the process of extracting features in the sliding window, to reduce the computational cost, we select five features in the time domain: MAV, slope sign change (SSC), waveform length (WL), root mean square (RMS), and Hjorth parameter (HP) [34].

a. MAV

The MAV is one of the most commonly used values in sEMG signal analysis. The MAV feature is the average of the absolute values of the amplitude of the sEMG signal in the sliding window. It provides information about the muscle contraction level. *s*(*k*) is the *k*th amplitude sample, *N* is the sample size. MAV can be calculated as *M*:

(1) $$M=\frac{1}{N}\sum_{k=1}^{N}\left|s\left(k\right)\right|$$

b. RMS

RMS represents the mean power of the sEMG signal, which reflects the activity of muscles. It is defined as *R* in Equation (2).

(2) $$R=\sqrt{\frac{1}{N}\sum_{k=1}^{N}s{\left(k\right)}^{2}}$$

c. SSC

SSC is another method of indicating the frequency information of the sEMG signal. It is defined as *S* in Equation (3) and represents the number of slope sign changes in the sliding window.

(3) $$S=\sum_{k=2}^{N-1}\left|\left(s\left(k\right)-s\left(k-1\right)\right)\times \left(s\left(k\right)-s\left(k+1\right)\right)\right|$$

d. WL

WL is the cumulative length of the sEMG signal waveform, which is related to waveform amplitude, frequency, and time and can be used to measure signal complexity. It is defined as *W* in Equation (4).

(4) $$W=\sum_{k=2}^{N}\left|s\left(k\right)-s\left(k-1\right)\right|$$

e. HP

HP was first used to analyze electroencephalographic signal in the time domain. It is composed of three parameters (activity, mobility, and complexity) based on variance calculation. 

The activity parameter can indicate the surface of the power spectrum in the frequency domain. It can be calculated as *A_hp_* by:

(5) $$A_{hp}=\mathrm{VAR}\left(s\left(k\right)\right)=\frac{1}{N-1}\sum_{k-1}^{N}s{\left(k\right)}^{2}$$

The estimate of the average frequency of the signal is usually expressed by the mobility parameter. It is calculated as *M_hp_*:

(6) $$M_{hp}=\sqrt{\frac{\mathrm{VAR}\left(\frac{ds\left(k\right)}{dk}\right)}{\mathrm{VAR}\left(s\left(k\right)\right)}}$$

The complexity parameter is the ratio of the mobility of the signal derivative to the mobility of the signal itself. The more similar the signal shape is to the pure sine wave, the closer the value is to 1. It is calculated as *C_hp_*:

(7) $$C_{hp}=\frac{\mathrm{Mobility}\left(\frac{ds\left(k\right)}{dk}\right)}{\mathrm{Mobility}\left(s\left(k\right)\right)}$$

To improve the accuracy of the classification, in addition to using the feature parameters described above, we also extract the preprocessed signals through the sliding windows, and put them together to form the feature matrix for the classifier.

#### 3.2.3. Classifier

In this work, we use a forward neural network which has three layers: the input layer, the hidden layer, and the output layer. The number of hidden layer nodes is taken to be half of the length of the feature vector. The output layer has six cells corresponding to the number of predicted gestures. The model uses a sigmoid transfer function. It is trained by using full batch gradient descent, with a cross entropy cost function.

#### 3.2.4. The ANN Classifier Training Algorithm

Based on the criteria above, the training algorithm (Algorithm 1) is described as follows.


**Algorithm 1**
**Input:** Standardized raw training data   $T=(T1,T2\cdots T8)\in {\left[-1,1\right]}^{N\times 8}$  Gesture labels  yi∈{0,1,2⋯c−1} **Output:**
  Trained ANN Classifier**Step 1** Preprocessing  **For** each user’s data **T**  **Do**
   $\Phi \left(T\right)\in {\left[-1,1\right]}^{m\times 8}$
△ muscle detection function Φ   $\mathrm{abs}\left[\Phi \left(T\right)\right]\in {\left[0,1\right]}^{l\times 8}$
△ absolute value function.    $M=\left(M_{1},M_{2}\cdots M_{8}\right)=\Gamma \left[\mathrm{abs}\left[\Phi \left(T\right)\right]\right]\in {\left[0,1\right]}^{l\times 8}$
△ 4th Butterworth filter Γ  **End** **End** **Step 2** Feature extraction  **For** each user’s data **T**  **Do**   $\;W_{j}={\left(M,R,S,W,A_{hp},\;M_{hp},C_{hp}\right)}^{T}\in {\mathbb{R}}^{7\times 1}$   $\;W=\left(W_{1},W_{2}\cdots W_{8}\right)\in {\mathbb{R}}^{7\times 8}$  **End**
  $D=\left(W,M\right)\in {\mathbb{R}}^{(l+7)\times 8}$
△ Feature vector D **End** **Step 3** Classification  **For** one user’s data **T**  Train the ANN Classifier with Features **D** and Gesture labels *y_i_* **End**


### 3.3. Testing Part

#### 3.3.1. Preprocessing and Feature Extraction

For the test set, we still use the absolute value function and the fourth-order Butterworth filter. In the feature extraction process, the five time-domain features (MAV, RMS, SSC, WL, HP) and preprocessed data are put together to form feature vectors.

We use the sliding window with the same length as in the training step to extract the feature vectors.

#### 3.3.2. Testing

In the process of testing, we obtain a vector of labels, where each label corresponds to the feature vector of a sliding window observation by the trained ANN classifier. Here, we apply an answering racer algorithm to assign a label to the test gesture.

For real-time processing, we define the classifier as $\Psi \left(Z_{i}\right)$. The return gesture $y_{i}\in \left\{0,1,2\cdots c-1\right\}$ is labeled by the ANN classifier from each observation window, where *c* denotes the number of gestures to recognize. Here, the label 0 represents the class “No-Gesture”. We define the classifier $\Psi :y_{i}\to Z_{i}$ in such a way that, we count the labels of each class returned separately, $N_{t}:=N_{t}+1$, that starts with $N_{t}=0$. the classifier is formulated as follows

(8) $$\Psi \left(Z_{i}\right)=\left\{ \begin{array}{l} t,\max\left\{N_{0},N_{1},\cdots N_{c-1}\right\}>\tau \\ 0,\max\left\{N_{0},N_{1},\cdots N_{c-1}\right\}<\tau \end{array}\right.$$

where t∈{0,1,2⋯c−1} denotes the recognized gesture. When meeting the condition that $N_{t}$ must be equal to or greater than the threshold τ, we think that the gesture is recognized, $\Psi (Z_{i})=t$. Otherwise, we define $\Psi \left(Z_{i}\right)=0$, which means that the classifier Ψ assigns $Z_{i}$ to the class “No-Gesture”. 

#### 3.3.3. Feature Extraction & Classification Algorithm

Based on the criteria above, the testing algorithm (Algorithm 2) is described as follows.


**Algorithm 2**
**Input:** Standardized raw testing data   $T=(T1,T2\cdots T8)\in {\left[-1,1\right]}^{N\times 8}$ **Output:** Final output gesture   $Z_{i}=t\in \left\{0,1,2\cdots c-1\right\}$
**Step 1** Preprocessing  **For** each user’s data **T**  **Do**
   $\Phi \left(T\right)\in {\left[-1,1\right]}^{m\times 8}$
△ muscle detection function Φ   $\mathrm{abs}\left[\Phi \left(T\right)\right]\in {\left[0,1\right]}^{l\times 8}$
△ absolute value function.   $M=\left(M_{1},M_{2}\cdots M_{8}\right)=\Gamma \left[\mathrm{abs}\left[\Phi \left(T\right)\right]\right]\in {\left[0,1\right]}^{l\times 8}$
△ 4th Butterworth filter Γ  **End** **End** **Step 2** Feature extraction  **For** each user’s data **T**  **Do**   $\;W_{j}={\left(M,R,S,W,A_{hp},\;M_{hp},C_{hp}\right)}^{T}\in {\mathbb{R}}^{7\times 1}$   $\;W=\left(W_{1},W_{2}\cdots W_{8}\right)\in {\mathbb{R}}^{7\times 8}$  **End**
  $D=\left(W,M\right)\in {\mathbb{R}}^{(l+7)\times 8}$
△ Feature vector D **End** **Step 3** Classification **For** each user’s data **T**  Using ANN classifier return gesture labels *y_i_*
  **the classifier**
$\Psi :y_{i}\to Z_{i}$, $N_{t}\in \left\{N_{0},N_{1},\cdots N_{c-1}\right\}$  **While** $N_{t}\leq \tau$   **Do**  $N_{t}:=N_{t}+1$  **End**  $\Psi \left(Z_{i}\right)=\left\{ \begin{array}{l} t,\max\left\{N_{0},N_{1},\cdots N_{c-1}\right\}>\tau \\ 0,\max\left\{N_{0},N_{1},\cdots N_{c-1}\right\}<\tau \end{array}\right.$ **End**

## 4. Experimental Results

In this section, our proposed method is evaluated on the real data from the MYO sensor. The data sets and the source codes are publicly available at the following link: https://github.com/yang kuoshu/https-github.com-yangkuoshu-Real-time-gesture-recognition-using-myo.git. The results are represented and compared with the results of other methods. At the same time, more detailed information is given to illustrate our approach. 

### 4.1. Recognition Accuracy

**Figure 6:**
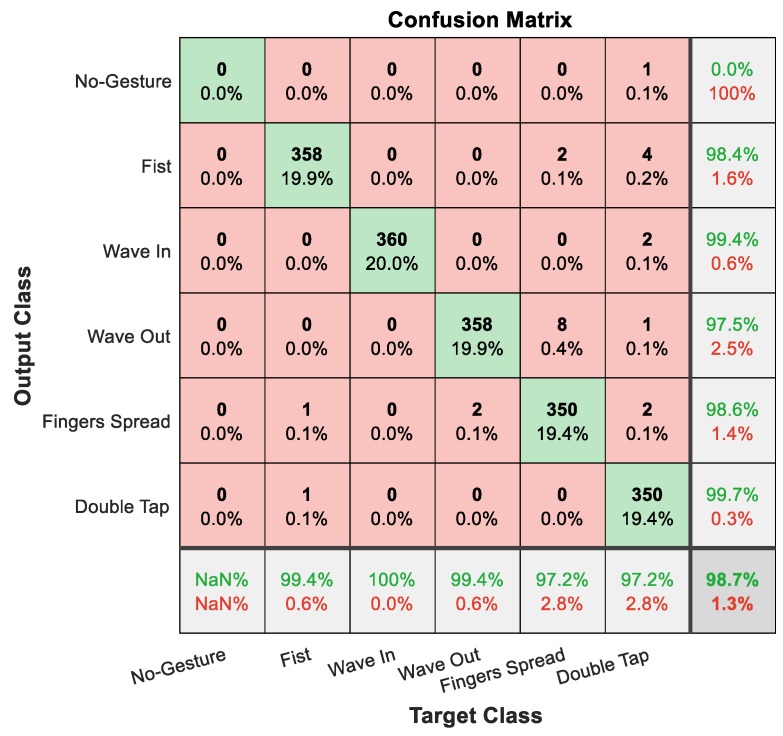
Confusion matrix of the results.

The confusion matrix for the proposed model is illustrated in Figure 6. This confusion matrix shows an overall recognition accuracy of 98.7%. The gesture “Wave In” is the one with the highest sensitivity (100%). The gestures “Fingers Spread” and “Double Tap” are both with the lowest (97.2%). Regarding precision, the gesture “Double Tap” has a near perfect result (99.7%) and the gesture “Wave Out” is the lowest (97.5%). Therefore, the proposed method easily mistakes the gesture “Fingers Spread” as the gesture “Wave Out”. Additionally, there is one repetition which is predicted as “No Gesture” because it cannot pass the threshold for preprocessing or postprocessing.

### 4.2. Real Time Pattern Recognition

**Figure 7:**
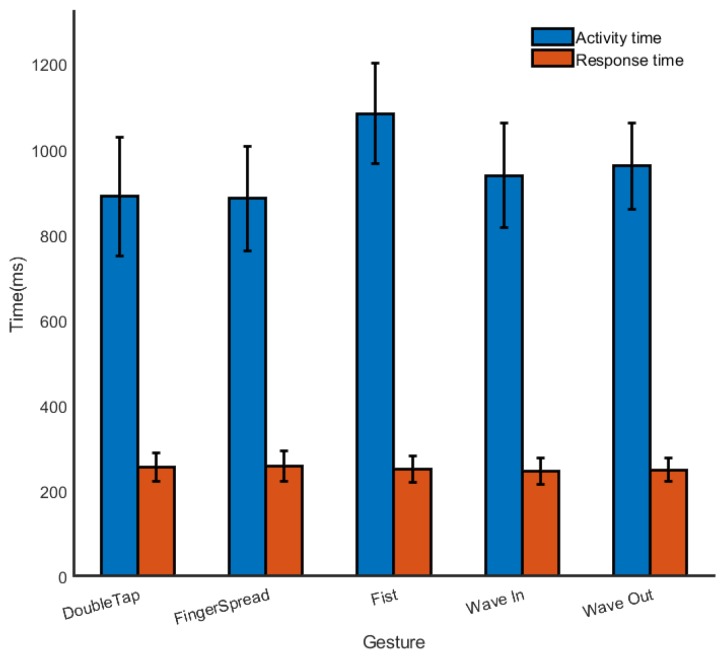
Average response time for each gesture.

As mentioned above, a method that is suitable for some applications requires not only high recognition accuracy but also real-time response. In reality, most pattern recognition methods start timing after the gesture is completed. Unlike other methods, our method starts timing at the beginning of the gesture. Figure 7 shows the average response times for each gesture (orange color). All response times are below 300 ms. Figure 7 also shows the response times of our method vs. the action times of all the gestures. Obviously, the response times are much smaller than action times, which means that the gesture can be recognized soon after the gesture begins. 

Table 1 shows the response times and recognition accuracies of all subjects. From the table, the recognition accuracy of all subjects except subject 9 can reach 98% or more. There are four subjects whose recognition accuracy can reach 100%. The average response times of all the subjects range from 187 ms to 293 ms. It shows that the proposed method has high recognition accuracy and short response time.

### 4.3. Sliding Windows Size

**Figure 8:**
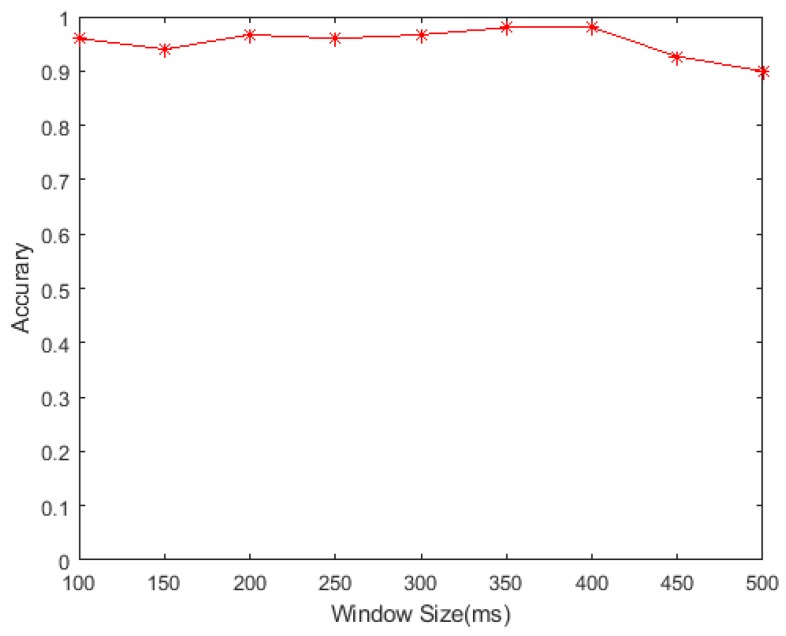
Window size test recognition rate (Subject 1).

To evaluate the effect of sliding window sizes in our method, we use different sliding window sizes in the process of gesture feature extraction for Subject 1. We test values of the window size from 100 ms to 500 ms (the step is 50 ms) as shown in Figure 8. It can be seen that the recognition rate has almost no increase when the window size is increased from 100 ms to 400 ms, and the recognition rate will decrease when the window size exceeds 400 ms. 

**Figure 9:**
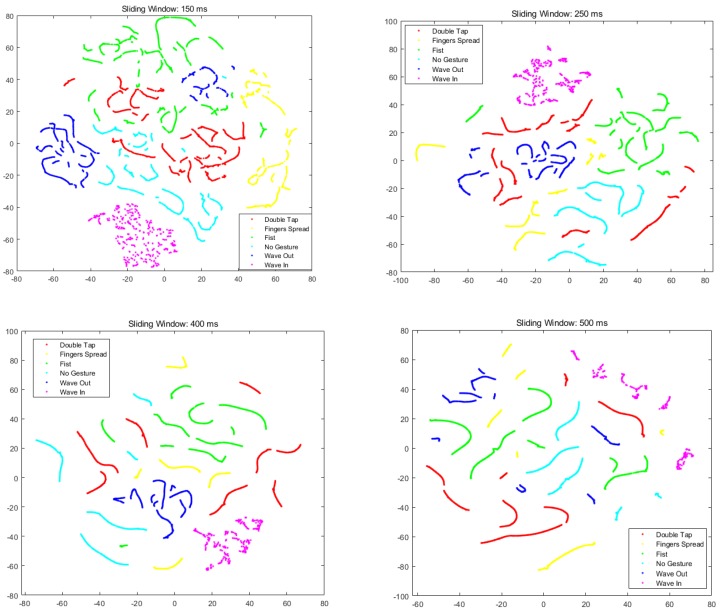
T-distributed stochastic neighbor embedding (t-SNE) results from different sliding window lengths.

To further determine an appropriate sliding window size, we used t-distributed stochastic neighbor embedding (t-SNE) to visualize how the feature vectors from each gesture class are clustered in the feature space [35]. The results from the t-SNE are shown in Figure 9. We can note that as the length of the sliding window increases, the projected feature vectors of each class get closer to each other. However, if the length of the sliding window increases beyond a certain level (here, this is 400 ms), it will result in the recognition method tending to overfit because the amount of feature vectors from a repetition is reduced and the length of the feature vector increases. Therefore, we set the sliding window size to 400 ms in our method.

### 4.4. Threshold of Activation Times

**Figure 10:**
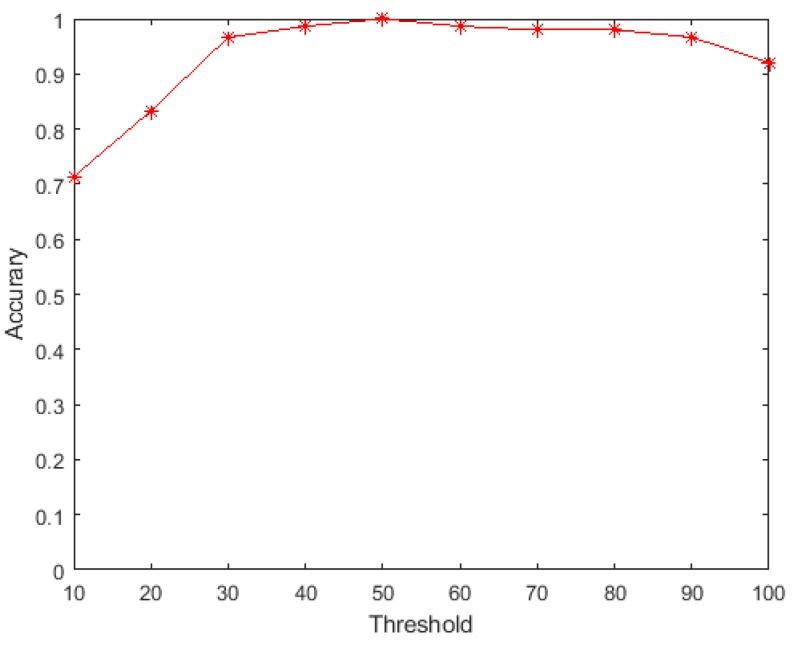
Thresholds in the method (Subject 1).

We also evaluated the effect of different classification thresholds of the activation times for Subject 1. As shown in Figure 10, when the threshold changes from 10 to 50, the recognition accuracy is rapidly increased. When the threshold is greater than 50, the recognition accuracy starts to decrease, and approaches 0.9 when the threshold increases to 100. If the threshold is too small, it will lead to misclassification. If the threshold is too large, it will cause the classifier to classify the gesture as “no gesture”, resulting in misclassification. In our approach, the smaller the threshold, the shorter the time required by our classifier; therefore, choosing an appropriately small threshold while maintaining accuracy could reduce the response time. Here, we set the activation time threshold to 40.

### 4.5. Comparison with Other Methods

Table 2 shows that the proposed model, which uses both types of features (the preprocessed signal values and the results from the bag of functions), has the best accuracy compared to the other models. The model that uses only the preprocessed signal values responds slower than the model that uses only the results from the bag of functions, but its recognition accuracy is higher. Table 2 also shows that the proposed model responds in 227.76 ms, which is lower than the real-time limit (300 ms). A comparison with other researchers’ methods in terms of response time and accuracy is presented, some of which are not real-time recognition models, so only their accuracy is shown here. It can be seen that, while guaranteeing real-time response, our proposed model has lower response time and higher accuracy.

Furthermore, compared with the existing hand gesture recognition models based on the ANN algorithm [24,39] which do not include the gesture duration in the eventual system response time, our proposed ANN pattern recognition system is not only capable of sharply reducing the total response time, but also lowers the computational load for the classifier by using the threshold of activation times.

## 5. Conclusions

In this paper, we have presented a real-time hand gesture recognition model based on sEMG signals. We use the MYO armband to acquire sEMG signals and apply a sliding window approach to segment the data for extracting features. A feedforward artificial neural network is founded and trained by the training dataset. A test method is used that the gesture will be recognized when recognized label times reach the threshold of activation times by the ANN classifier. The model responds in 227.76 ms from the beginning of the gesture, which is lower than the limit defined for real time (300 ms). At the same time, the model shows a recognition accuracy of 98.7%, which is higher than the state-of-the-art. 

## Figures and Tables

**Table 1:** Recognition rate and response time of all subjects.

Subject	Accuracy (%)	Response (ms)
Subject 1	98.67	214.73
Subject 2	100.00	212.40
Subject 3	98.00	205.43
Subject 4	100.00	291.10
Subject 5	100.00	292.20
Subject 6	100.00	244.40
Subject 7	99.33	215.73
Subject 8	99.33	193.23
Subject 9	93.33	233.37
Subject 10	98.00	232.40
Subject 11	98.00	210.87
Subject 12	99.33	187.23

**Table 2:** The proposed model compared with other models.

Model	Accuracy (%)	Response (ms)
Evaluated models:		
Proposed model	98.7	227.76
Model using only the preprocessed signals values	96.0	238.03
Model only using only the results from the bag of functions	86.0	227.63
Other methods with MYO armband sensors		
MYO armband method [24]	83.1	X
Model using k-NN with DTW [11,24]	89.5, 90.54	X
Model using SVM [11,36]	92, 93.99	X
Model using ANN [24]	90.7	X
Model using Discriminant Analysis [37]	94.54	X
Model using Naive Bayes [37]	81.76	X
Model using Random Forest [37]	89.92	X
Model using deep learning [38]	98.31	X
